# A Comprehensive Review on Managing Fracture Calcaneum by Surgical and Non-surgical Modalities

**DOI:** 10.7759/cureus.54786

**Published:** 2024-02-23

**Authors:** Aditya Chirayath, Nareshkumar Dhaniwala, Kevin Kawde

**Affiliations:** 1 Department of Orthopaedic Surgery, Jawaharlal Nehru Medical College, Datta Meghe Institute of Higher Education and Research, Wardha, IND

**Keywords:** tele-rehabilitation, multidisciplinary collaboration, patient compliance, rehabilitation strategies, surgical modalities, calcaneal fractures

## Abstract

This comprehensive review delves into the multifaceted landscape of calcaneal fractures, thoroughly examining their aetiology, clinical presentation, and diverse management strategies. Encompassing surgical and non-surgical approaches, the review scrutinises critical aspects such as patient compliance, rehabilitation protocols, and long-term follow-up considerations. Surgical modalities, propelled by recent innovations like minimally invasive techniques and advanced fixation materials, are juxtaposed with non-surgical interventions, emphasising the pivotal role of patient education and adherence to optimise outcomes. The synthesis of critical findings underscores the need for individualised care and multidisciplinary collaboration in clinical practice. Moreover, the review outlines recommendations for healthcare practitioners and identifies promising areas for future research, including biomechanical studies and telerehabilitation. This comprehensive exploration aims to contribute to the ongoing evolution of calcaneal fracture management, ultimately enhancing patient care and outcomes in this complex orthopaedic realm.

## Introduction and background

Calcaneal fractures involving the heel bone represent a complex orthopaedic challenge due to the intricate anatomy and the pivotal role of the calcaneus in weight-bearing. Typically resulting from high-energy trauma such as falls from height or motor vehicle accidents, these fractures can significantly impact a patient's quality of life. The calcaneus is a critical component of the foot's arch and is crucial in maintaining stability during ambulation. Understanding the nuances of calcaneal fractures is paramount for clinicians involved in their management [1].

The annual incidence of calcaneal fractures is approximately 11.5 per 100,000 individuals, with a significantly higher occurrence in males (16.5 per 100,000) than females. Falls from a height are the most common cause of calcaneal fractures, accounting for approximately 47.5% of cases. Calcaneal fractures are relatively uncommon, comprising 1 to 2% of all fractures [2]. Effective management of calcaneal fractures is imperative to mitigate complications and ensure optimal functional outcomes. The intricate interplay of subtalar joint function, soft tissue integrity, and the complex anatomy of the calcaneus necessitates a comprehensive approach. Neglected or improperly managed calcaneal fractures can lead to persistent pain, altered gait, and long-term disability. Hence, a nuanced understanding of surgical and non-surgical interventions is crucial for healthcare professionals treating calcaneal fractures [2].

The purpose of this review is to provide a thorough examination of the various modalities available for the management of calcaneal fractures. By synthesising current literature, we aim to offer insights into clinical presentation, diagnostic considerations, and treatment options, including surgical and non-surgical approaches. This comprehensive exploration seeks to equip healthcare practitioners with a nuanced understanding of the complexities associated with calcaneal fractures, facilitating informed decision-making in pursuing optimal patient outcomes. Through this review, we aspire to contribute to the evolving landscape of calcaneal fracture management, fostering a holistic and evidence-based approach in clinical practice.

## Review

Etiology of calcaneal fractures

Common Causes

Falls from height: Falls from elevated surfaces, such as ladders or roofs, represent a significant mechanism of injury for calcaneal fractures. The impact force generated upon landing can exert substantial pressure on the calcaneus, resulting in fractures. Individuals involved in occupations or activities that require working at heights face an increased risk, making fall prevention measures and safety protocols crucial in mitigating such incidents [3].

Motor vehicle accidents: In the context of motor vehicle accidents, collisions can have severe implications for the calcaneus, especially when the foot experiences direct impact or crushing forces. High-speed accidents amplify the trauma, leading to fractures. Understanding the dynamics of these accidents and implementing preventive measures, such as proper vehicle safety features and awareness campaigns, becomes essential in reducing the incidence of calcaneal fractures in these scenarios [4].

Sports-related injuries: Certain sports, particularly those involving contact or rapid changes in direction, pose a heightened risk of traumatic foot injuries, including calcaneal fractures. Athletes engaged in activities such as football, basketball, or gymnastics may be susceptible. Injury prevention strategies, such as proper training techniques, equipment, and awareness programs, are integral in minimising the risk of sports-related calcaneal fractures [5].

Crush injuries: Occupational settings involving heavy machinery or equipment present a specific risk for crush injuries to the calcaneus. Accidents where the foot is subjected to crushing forces can result in fractures. Occupational safety measures, including training, proper use of protective gear, and machinery safety protocols, are imperative to reduce the occurrence of crush injuries and subsequent calcaneal fractures in workplaces with these hazards [6].

Mechanism of Calcaneal Fracture

The mechanism of injury typically involves a high-energy axial load applied to the heel, which drives the talus downward onto the calcaneus. A combination of shear and compression forces produces two characteristic primary fracture lines. Shearing forces produce a fracture dividing the calcaneus into medial and lateral portions. Compression forces divide the calcaneus into anterior and posterior portions. This fracture line can extend medially to involve the middle facet. Loss of calcaneal height and length are readily explained by this mechanism [3].

Risk Factors

Osteoporosis: Osteoporosis, characterised by reduced bone density and compromised bone strength, significantly heightens the vulnerability to fractures, particularly in the elderly. As a weight-bearing bone, the calcaneus becomes particularly susceptible to fractures due to osteoporosis. Preventive measures, including bone density assessments, dietary interventions, and appropriate medical management, are crucial in mitigating the risk of calcaneal fractures in individuals with osteoporosis [7].

Occupational hazards: Professions that involve extended periods of standing, heavy lifting, or exposure to potential foot injuries pose a heightened risk of calcaneal fractures. Workers in construction, manufacturing, or other physically demanding occupations may be particularly vulnerable. Implementing workplace safety measures, providing proper footwear, and educating employees on injury prevention contribute to reducing the occupational hazards associated with calcaneal fractures [8].

Age and gender: Younger individuals, especially males, face an elevated risk of calcaneal fractures due to increased participation in activities with a higher potential for trauma. The propensity to engage in physically demanding or high-impact activities increases the risk. Tailoring preventive strategies, such as targeted educational campaigns and safety guidelines, to this demographic is essential in reducing the incidence of calcaneal fractures in these populations [9].

Medical conditions: Certain conditions, such as peripheral or diabetic neuropathy, compromise foot sensation, leading to an increased risk of falls and subsequent calcaneal fractures. Individuals with these conditions may not perceive potential hazards or uneven surfaces, contributing to an elevated risk. Routine foot examinations, sensory testing, and fall prevention strategies are paramount in managing the heightened risk associated with specific medical conditions and reducing the incidence of calcaneal fractures in affected individuals [10].

Types of Calcaneal Fractures

Intra-articular fractures: Intra-articular fractures involve the extension of the fracture into the subtalar joint, significantly impacting joint congruity and function. These fractures often present complex challenges, directly affecting the articulation between the calcaneus and surrounding bones. Management of intra-articular fractures requires meticulous attention to anatomical alignment to preserve joint function and surgical intervention may be indicated to achieve optimal outcomes [11].

Extra-articular fractures: Extra-articular fractures, in contrast, do not involve the subtalar joint and typically affect the body or process of the calcaneus. While they may be less complex than intra-articular fractures, they require careful evaluation and management. The absence of joint involvement may influence treatment choice, with non-surgical approaches being more feasible in some instances [12].

Closed fractures: Closed fractures are characterised by an intact skin surface, reducing the risk of infection compared to open fractures. Despite the lower risk of infection, careful management is essential, involving accurate diagnosis through imaging, immobilisation, and adherence to rehabilitation protocols. The potential for complications, such as delayed union or nonunion, necessitates close monitoring and appropriate intervention to facilitate optimal healing [12].

Open fractures: Open fractures, marked by a break in the skin, pose a heightened risk of infection and often demand urgent attention. The exposure of the fracture site to external contaminants necessitates prompt wound management, debridement, and administration of antibiotics. Surgical intervention is often indicated to address both the fracture and the associated soft tissue injuries. Open fractures require a comprehensive and coordinated approach to minimise the risk of complications and optimise the chances of successful healing [12].

Clinical presentation

Signs and Symptoms

Pain: Pain is a hallmark symptom of calcaneal fractures, characterised by acute and localised discomfort at the heel. This pain is often intensified during weight-bearing activities, reflecting the stress placed on the fractured calcaneus. The severity of pain can vary depending on the extent and type of fracture, influencing both the diagnosis and subsequent management strategies aimed at pain relief and functional recovery [13].

Swelling: Edema, or swelling, is a common clinical manifestation observed around the heel and the lateral aspect of the foot in cases of calcaneal fractures. The presence of swelling indicates soft tissue involvement and is a critical clinical sign used in conjunction with other symptoms and diagnostic tools to assess the extent of the injury. Effective management often includes interventions to address and reduce this swelling, improving patient comfort and recovery [13].

Ecchymosis: Bruising, or ecchymosis, may develop in the presence of associated soft tissue injury accompanying calcaneal fractures. The discolouration results from blood leakage into the surrounding tissues, providing valuable visual cues for healthcare professionals during clinical assessment. The severity and distribution of ecchymosis contribute to the overall understanding of the injury and guide appropriate treatment strategies [13].

Deformity: In more severe calcaneal fractures, visible deformity or widening of the heel may be observed. This deformity is indicative of significant displacement or disruption of the calcaneal anatomy. Recognising and assessing deformities are crucial in diagnosing and classifying fractures and guiding treatment decisions towards interventions that restore proper anatomical alignment [13].

Impaired function: Calcaneal fractures often lead to impaired function, with patients having trouble walking and noticeable alterations in gait and weight-bearing. The extent of functional impairment varies depending on the severity and type of fracture. Comprehensive rehabilitation strategies, including physiotherapy and targeted exercises, are crucial components of the treatment plan to address impaired function and facilitate a gradual return to normal activities [13].

Diagnostic Tools and Imaging Techniques

In addition to standard radiographs, which include anteroposterior (AP) and lateral views, diagnostic imaging for calcaneal fractures should encompass assessment for specific radiological signs such as Mondor's Sign, Bohler's Angle, and the Critical Angle of Gissane. These signs offer valuable insights into fracture patterns and associated soft tissue injuries, aiding in accurate diagnosis and treatment planning [3].

X-ray imaging: X-ray imaging is a foundational diagnostic tool for evaluating calcaneal fractures. Alongside standard radiographs, oblique views may enhance the visualisation of fracture patterns not clearly discernible in standard projections. X-rays play a crucial role in guiding treatment decisions and determining the appropriate management course by providing initial insights into the fracture extent and configuration [3].

Computed tomography (CT) scans: CT scans provide a more detailed and three-dimensional assessment of calcaneal fractures, facilitating thorough evaluation of intra-articular involvement, fracture displacement, and comminution. CT imaging offers a comprehensive visualisation of the bony structures, aiding orthopaedic surgeons in surgical planning and providing a more precise understanding of the fracture's complexity [3].

Magnetic resonance imaging (MRI): MRI is indispensable for assessing soft tissue injuries associated with calcaneal fractures, including ligamentous involvement and subtalar joint damage. By offering detailed images of soft tissues, MRI complements information obtained from X-rays and CT scans, providing a comprehensive understanding of the injury's extent, particularly beyond the bony structures [3].

Ultrasonography: Although less commonly utilised for calcaneal fractures, ultrasonography can effectively assess soft tissue injuries and hematoma formation. This imaging modality utilises sound waves to generate real-time images, providing dynamic visualisation of the soft tissues surrounding the calcaneus. While less frequently employed than X-rays, CT scans, or MRI, ultrasonography may offer valuable insights into soft tissue components of the injury in specific cases [3]. Additionally, the assessment of Mondor's Sign, Bohler's Angle, and the Critical Angle of Gissane should be incorporated into the diagnostic protocol to ensure comprehensive evaluation and accurate diagnosis of calcaneal fractures. Classification systems for calcaneal fractures are described in Table 1 [14].

**Table 1:** Sanders classification and computed tomography classification (Essex-Lopresti) for calcaneal fractures Source: [14]

Classification	Description
Sanders classification	
Type I	Avulsion fractures without joint involvement.
Type II	Fractures involving the joint surface without displacement.
Type III	Joint depression fractures with various degrees of displacement.
Type IV	Split fractures involving the anterior and posterior facets.
Computed tomography classification (Essex-Lopresti)	
Joint depression (A)	It involves the subtalar joint.
Tongue-type (B)	Fracture extends anteriorly into the anterior calcaneal process.
Joint depression and tongue-type (C)	Combination of A and B patterns.

Non-surgical modalities

Rest, Ice, Compression, and Elevation (RICE)

Rest: Rest is the cornerstone of the initial management of calcaneal fractures, aiming to minimise weight-bearing on the affected foot. By reducing mechanical stress on the fractured calcaneus, rest provides the necessary conditions for the bone to heal. Immobilisation, often achieved through the use of crutches or a brace, serves to prevent further trauma to the injured area. This critical phase of rest allows for the initial stages of healing and sets the foundation for subsequent rehabilitation [1-2].

Ice: Cold therapy, in the form of ice application, is integral in managing pain and inflammation associated with calcaneal fractures. Applying ice packs to the affected area in 20-minute intervals helps constrict blood vessels, reducing blood flow to the injured site and alleviating pain. Cold therapy is particularly effective in the early stages post-injury, contributing to the patient's overall comfort and aiding in controlling inflammatory responses [2].

Compression: Compression controls swelling and supports the injured foot. Compression bandages or wraps help minimise oedema by exerting pressure on the tissues, preventing excessive fluid accumulation. It is essential, however, to apply compression carefully to avoid excessive pressure that may compromise blood circulation. Properly applied compression contributes to the overall stability of the injured area and assists in creating a conducive environment for healing [3].

Elevation: Elevating the foot above heart level, especially during rest periods, is a simple yet effective measure to minimise swelling associated with calcaneal fractures. By facilitating fluid drainage and reducing the pooling of blood and other fluids in the injured region, elevation complements the actions of rest, ice, and compression. This elevation strategy is often recommended as part of the initial management protocol and aids in creating an environment conducive to optimal healing [1].

Analgesic and Anti-Inflammatory Medications

Nonsteroidal anti-inflammatory drugs (NSAIDs): NSAIDs represent a pharmacological approach to pain management and inflammation reduction in the context of calcaneal fractures. Medications such as ibuprofen or naproxen are often prescribed to alleviate pain by inhibiting the production of prostaglandins, which contribute to pain and inflammation. While NSAIDs are effective, caution is advised due to potential side effects such as gastrointestinal irritation, renal issues, and interactions with other medications. Healthcare providers carefully consider the patient's medical history, existing conditions, and the potential for adverse effects before recommending NSAIDs [15].

Pain management: Analgesics, including acetaminophen, are employed for pain control in individuals with calcaneal fractures, particularly in cases where NSAIDs may be contraindicated or not well-tolerated. Acetaminophen functions centrally to reduce pain perception and is often chosen when anti-inflammatory effects are not the primary focus. As with any medication, appropriate dosage and adherence to recommended guidelines are essential to mitigate the risk of adverse effects. Pain management strategies are tailored to the individual patient, considering factors such as pain intensity, overall health, and potential drug interactions [16].

Physiotherapy and Rehabilitation

Range of motion exercises: One key component of physiotherapy involves a range of motion exercises aimed at enhancing flexibility without exerting undue pressure on the healing heel. These exercises include gentle toe flexion and extension movements, fostering toe flexibility while mitigating strain on the injured area. Additionally, controlled ankle circles are employed to maintain mobility in the ankle joint, contributing to overall joint health during the recovery phase [17].

Strengthening exercises: Strengthening exercises are integral to the rehabilitation program, targeting the gradual restoration of muscle strength. Calf raises are fundamental exercises involving the heel's incremental elevation while standing, effectively engaging and strengthening the calf muscles. Seated toe-tapping exercises provide a rhythmic and controlled method to enhance foot and ankle strength, improving overall lower extremity function [18].

Weight-bearing progression: The progression of weight-bearing activities is carefully managed to prevent muscle atrophy and facilitate optimal healing. Physiotherapists introduce partial weight-bearing activities tailored to patients' tolerance to prevent disease-related complications. As healing advances, a progressive weight-bearing approach is implemented, with the physiotherapist guiding the sequential increase in weight-bearing activities. This stepwise progression aligns with the individual's healing trajectory, ensuring a balance between promoting recovery and minimising the risk of complications [19].

Surgical modalities

Indications for Surgery

Joint involvement: Fractures with significant intra-articular displacement, especially those impacting joint surfaces, may necessitate surgical intervention. Joint involvement poses a risk of altered joint congruity, which, if not addressed, could lead to long-term arthritis and functional impairment. Surgery, in these cases, aims to restore joint alignment and minimise the risk of degenerative changes, ultimately preserving joint function and minimising post-traumatic complications [20].

Severe displacement: Calcaneal fractures demonstrating substantial displacement, particularly when involving the subtalar joint, often require surgical reduction and fixation. The severity of displacement can compromise anatomical alignment, affecting the foot biomechanics. Surgical interventions, such as open reduction and internal fixation, are employed to realign the fractured fragments and stabilise the calcaneus. This surgical approach optimises long-term outcomes by addressing the significant displacement observed in severe fractures [11].

Open fractures: Surgical intervention is frequently indicated for open fractures, with a break in the skin. Open fractures pose an increased risk of infection due to exposure of the fracture site to external contaminants. Surgical procedures are performed to reduce the risk of infection, facilitate proper wound management, and stabilise the fractured calcaneus. Swift intervention is crucial to minimise complications associated with open fractures and promote optimal healing [21].

Intra-articular step-offs: The presence of step-offs or gaps in the articular surface, particularly those affecting joint function and stability, may necessitate surgical correction. Intra-articular step-offs can disrupt joint congruity, leading to impaired joint mechanics and potential post-traumatic arthritis. Surgical techniques, including precision reduction and fixation, restore the articular surface and prevent long-term joint dysfunction. Addressing intra-articular step-offs is paramount to achieving optimal functional outcomes in calcaneal fractures [22].

Timing of Surgery

Early surgical intervention: Early surgical intervention may be deemed necessary in specific cases, particularly those involving open fractures or instances with vascular compromise. Open fractures, where the skin is disrupted, pose an increased risk of infection and require prompt attention to address bone and soft tissue injuries. Additionally, cases with vascular compromise demand immediate surgical measures to restore blood supply and prevent further complications. Early surgery in these situations aims to mitigate the risks associated with open fractures and vascular compromise, facilitating optimal wound management and reducing the likelihood of postoperative complications [23].

Delayed surgery: In contrast, there are situations where a delayed surgical approach is preferred. Delayed surgery involves waiting until the initial soft tissue swelling and inflammation have subsided. This delay allows for improved surgical site visualisation, facilitating more precise interventions and reducing the risk of complications related to excessive soft tissue tension during surgery. Opting for delayed surgery can contribute to better wound healing, decreased postoperative swelling, and reduced potential complications. This approach is often considered when the immediate surgical risks associated with soft tissue compromise are outweighed by the benefits of a delayed, more controlled intervention [24].

Surgical Approaches

Open reduction and internal fixation (ORIF): ORIF is a critical surgical intervention for calcaneal fractures, involving precise fracture reduction followed by internal stabilisation. Two primary approaches are commonly employed in ORIF procedures [25]. The lateral extensile approach is a widely used method featuring a lateral incision that provides direct access to the calcaneus. This approach allows for a comprehensive view of the fracture site, facilitating meticulous reduction and stabilisation of the bone fragments. The advantages of the lateral extensile approach lie in its exposure capabilities, enabling surgeons to achieve accurate fixation and alignment during the surgical procedure [25]. In contrast, the sinus tarsi approach is a more limited lateral approach designed to minimise soft tissue disruption, often preferred for intra-articular fractures. By carefully navigating the sinus tarsi, surgeons can access and manage fractures within the subtalar joint. This approach aims to preserve surrounding tissues, providing a more conservative option while addressing fractures within the subtalar joint [25].

Percutaneous Fixation Techniques

Percutaneous fixation techniques offer minimally invasive alternatives for stabilising calcaneal fractures, providing benefits such as reduced soft tissue trauma and expedited recovery. Two prevalent percutaneous techniques include [26]. Percutaneous screw fixation involves the insertion of screws through small incisions directly into the fractured segments of the calcaneus. This minimally invasive technique aims to achieve stability and alignment without requiring extensive incisions. Percutaneous screw fixation is particularly advantageous for fractures amenable to this approach, offering benefits such as decreased soft tissue trauma and potentially faster postoperative recovery [27]. On the other hand, external fixation entails the application of external frames to maintain alignment and stability in the fractured calcaneus. This technique is often employed in complex fractures or situations where internal fixation is not optimal. The external frame supports and allows controlled adjustments, contributing to overall stability. While involving the placement of pins through the skin into the bone, external fixation proves advantageous in specific clinical scenarios [28].

Complications Associated with Surgical Interventions

Infection: Surgical procedures, especially in the context of open fractures, carry an inherent risk of infection. Open fractures involve a break in the skin, exposing the fracture site to external contaminants. Infections can compromise the healing process and may necessitate additional interventions, such as debridement or antibiotic therapy, to manage and mitigate the risk of systemic spread. Vigilant postoperative care and monitoring are crucial to identify and address infections promptly [23].

Wound healing issues: Impaired wound healing or skin necrosis can be complications associated with surgical intervention, particularly when extensive soft tissue dissection is required. Factors such as compromised blood supply, pre-existing medical conditions, or wound site infection can contribute to delayed wound healing. Close postoperative monitoring and wound care are essential to identify and address issues promptly, minimising the risk of further complications [29].

Hardware-related complications: The use of hardware in surgical fixation, such as screws or plates, can lead to complications such as loosening, migration, or irritation. Hardware-related issues may necessitate revision surgeries to address instability or discomfort. Regular follow-up assessments, imaging studies, and patient feedback are crucial in identifying and managing hardware-related complications to ensure the stability and effectiveness of the fixation [30].

Post-traumatic arthritis: Despite surgical intervention, some patients may develop post-traumatic arthritis, especially when there is significant joint involvement. Arthritic changes can occur over time, affecting joint function and causing pain. This long-term complication may require ongoing management, including pain control measures, lifestyle modifications, or, in severe cases, joint replacement surgery [31].

Neurovascular compromise: Nerve or vascular injuries may occur during surgery, leading to sensory or circulatory deficits. Careful preoperative planning, intraoperative monitoring, and surgical precision are essential to minimise the risk of neurovascular compromise. Postoperative assessments for signs of nerve damage or vascular compromise are crucial, and prompt intervention may be necessary to address these complications and optimise patient outcomes [32].

Comparative analysis

Pros and Cons of Surgical vs. Non-surgical Approaches

The decision to pursue surgical or non-surgical treatment for calcaneal fractures should be made on a case-by-case basis, considering the fracture's specific characteristics and the patient's circumstances. Some studies suggest that surgical treatment may lead to better recovery of the Bohler's Angle and more stable calcaneal height, while others report conflicting results [33-35]. It is essential to weigh the potential benefits of surgical intervention against the risks, including infection, nerve damage, and prolonged recovery time [33,35]. Non-surgical approaches may be associated with a lower risk of complications. Still, they may not always result in optimal anatomical and functional outcomes, especially in severe displacement or intra-articular involvement [34]. The functional outcomes of surgical versus non-surgical treatment for calcaneal fractures remain a topic of debate. While some studies suggest that surgical treatment may lead to better anatomical restoration and functional outcomes, others report conflicting results [33-35]. It is essential to consider the specific characteristics of the fracture, the patient's circumstances, and their functional goals when making treatment decisions.

Risk-Benefit Considerations

The decision to pursue surgical or non-surgical treatment for calcaneal fractures should consider each approach's potential risks and benefits. Surgical treatment may be associated with a higher risk of complications, such as infection, nerve damage, and prolonged recovery time. Still, it may also lead to better anatomical restoration and functional outcomes in some instances [33,35]. Non-surgical approaches may be associated with a lower risk of complications. Still, they may not always result in optimal anatomical and functional outcomes, especially in severe displacement or intra-articular involvement [34]. Therefore, the decision should be individualised based on the specific characteristics of the fracture and the patient's goals and preferences.

Postoperative care

Rehabilitation Protocols After Surgery

Early mobilisation: Early mobilisation is a critical component of postoperative care, aiming to prevent stiffness and promote joint function. Initiating mobilisation within the limits of surgical fixation helps maintain joint flexibility and prevent the development of contractures. Early mobilisation may initially involve non-weight-bearing or partial weight-bearing activities, gradually progressing as the patient's recovery allows. This proactive approach supports a quicker return to functional activities and helps prevent complications associated with prolonged immobilisation [36].

Physiotherapy sessions: Structured physiotherapy sessions are integral to the postoperative care plan for calcaneal fractures. These sessions address specific rehabilitation goals, focusing on restoring range of motion, building strength, and improving functional mobility. Physiotherapists tailor exercises to the individual patient's needs, taking into account the type and severity of the fracture and the surgical interventions performed. Regular physiotherapy promotes optimal recovery and ensures the patient achieves the milestones for successful rehabilitation [17].

Protected weight-bearing: The transition from non-weight-bearing to partial weight-bearing and eventually full weight-bearing is a gradual process guided by the treating orthopaedic team. Protected weight-bearing protocols are implemented to avoid excessive stress on the surgically reconstructed calcaneus during the initial phases of rehabilitation. This cautious approach minimises the risk of complications and supports the healing process. Close collaboration between patients and healthcare providers is crucial to ensure adherence to weight-bearing restrictions and promote a safe recovery [19].

Gait training: Gait training is essential to rehabilitation following calcaneal fracture surgery. Ensuring a proper and efficient walking pattern prevents gait abnormalities and associated complications. Gait training focuses on restoring a typical walking pattern, optimising weight distribution, and promoting balance. This aspect of rehabilitation plays a vital role in facilitating a smooth transition from protected weight-bearing to full weight-bearing. It ultimately enables patients to regain confidence in their walking abilities [37].

Monitoring and adjusting rehabilitation plan: Regular monitoring of the patient's progress allows for adjustments to the rehabilitation plan based on individual responses and healing trajectories. Healthcare providers assess pain levels, range of motion, and strength during follow-up appointments. Modifications to the rehabilitation plan may be made to address any challenges or to advance the intensity of exercises as the patient's condition improves. This dynamic and personalised approach ensures that the rehabilitation plan remains aligned with the patient's evolving needs, optimising the chances of a successful recovery [38].

Monitoring for Complications

Wound care: Vigilant wound care is paramount in the postoperative period following calcaneal fracture surgery. Continuous monitoring of the surgical incision site is essential to detect any signs of infection, delayed healing, or other wound-related issues. This includes assessing for redness, swelling, increased warmth, or discharge. Timely intervention in response to any concerning signs ensures proper wound healing and reduces the risk of complications [39].

Hardware integrity: Regular assessment of the stability and integrity of the fixation hardware is crucial to identify any signs of loosening or complications. This involves reviewing imaging studies, such as X-rays, to evaluate the positioning and condition of screws, plates, or other fixation devices. Monitoring hardware integrity helps prevent potential issues related to instability, malunion, or irritation, ensuring the effectiveness of the surgical reconstruction [40].

Neurovascular checks: Ongoing neurovascular checks involve monitoring for changes in the affected foot's sensation, circulation, or motor function. These checks are essential to detect potential neurovascular complications, such as nerve impingement or vascular compromise, which can occur during or after surgery. Regular assessments contribute to the early identification of any abnormalities, allowing for prompt intervention to prevent further complications [41].

Inflammatory response: Monitoring for excessive inflammation or signs of a postoperative inflammatory response is crucial. Persistent or worsening inflammation may indicate complications such as infection or an adverse reaction to hardware. If necessary, regular clinical assessments and laboratory tests help healthcare providers gauge the inflammatory status and take appropriate measures to address any underlying issues [42].

Functional progress: Regular evaluation of the patient's functional progress is integral to assessing the effectiveness of rehabilitation. This includes monitoring the range of motion, strength, and gait. Tracking functional improvements helps ensure steady progress and identifies any setbacks or challenges that may require adjustments to the rehabilitation plan. This comprehensive assessment supports achieving optimal functional outcomes for patients recovering from calcaneal fracture surgery [43].

Long-Term Follow-Up Considerations

Imaging studies: Periodic imaging studies, such as X-rays or CT scans, play a crucial role in the long-term follow-up of patients who have undergone calcaneal fracture surgery. These studies are conducted to assess the healing progress of the fracture, identify any hardware-related issues such as loosening or migration, and monitor for the development of post-traumatic arthritis. Regular imaging provides valuable insights into the structural integrity of the calcaneus and assists healthcare providers in making informed decisions regarding ongoing management or potential interventions [3].

Functional assessments: Long-term follow-up should include functional assessments to gauge the patient's ability to perform daily activities and participate in recreational or vocational pursuits. Functional assessments thoroughly evaluate a range of motion, strength, gait, and overall mobility. Monitoring functional outcomes helps healthcare providers tailor rehabilitation strategies, address persistent functional limitations, and optimise the patient's overall quality of life in the extended postoperative period [44].

Pain management: Continued monitoring and management of pain, if present, remain essential components of long-term follow-up. Chronic pain issues related to the calcaneal fracture or its surgical intervention may persist, impacting the patient's daily activities and overall well-being. Ongoing pain management strategies, including medication adjustments, physical therapy, or other interventions, aim to enhance the patient's quality of life and provide sustained relief from any lingering discomfort [45].

Patient education: Providing ongoing education to the patient is crucial during long-term follow-up. This involves discussing and reinforcing long-term expectations, potential limitations, and strategies for maintaining foot health. Patient education contributes to informed decision-making, encourages adherence to recommended lifestyle modifications, and empowers individuals to participate actively in their ongoing care. Clear communication about the anticipated trajectory of recovery and potential challenges enhances the patient's understanding and involvement in the long-term management process [46].

Screening for complications: Long-term follow-up includes vigilant screening for potential complications that may arise after calcaneal fracture surgery. This involves assessing for conditions such as osteomyelitis, arthritis, or malunion. Early identification of complications enables timely intervention and appropriate management strategies. Regular screenings may include clinical examinations, imaging studies, and other diagnostic tests, allowing healthcare providers to address complications promptly and optimise long-term outcomes for patients [47].

Recent advances and emerging technologies

Innovations in Surgical Techniques

Minimally invasive surgery (MIS): Advances in MIS techniques for calcaneal fractures represent a paradigm shift in surgical intervention approaches. Compared to traditional open procedures, MIS minimises soft tissue trauma, reduces scarring, and expedites recovery. Percutaneous fixation methods, including arthroscopy-assisted procedures, are at the forefront of MIS for calcaneal fractures. These techniques involve smaller incisions, allowing specialised instruments and implants to be inserted with the guidance of imaging technologies. By reducing the extent of soft tissue disruption, MIS endeavours to achieve stable fracture fixation while potentially decreasing complications associated with open procedures [48].

Computer-assisted surgery (CAS): Integrating CAS has significantly enhanced the precision and accuracy of surgical interventions for calcaneal fractures. CAS involves using navigation systems that provide real-time feedback to surgeons during the procedure. This technology aids in fracture reduction and implant placement, improving the overall accuracy of the surgical process. By enhancing the surgeon's ability to navigate complex anatomical structures, CAS contributes to more precise interventions, potentially reducing the risk of complications and optimising the outcomes of calcaneal fracture surgeries [49].

3D printing technology: 3D printing technology has introduced a personalised dimension to the management of calcaneal fractures. This innovative approach allows for the creation of patient-specific implants and surgical guides. By leveraging detailed imaging data, surgeons can design implants tailored to the patient's unique anatomy. Patient-specific surgical guides assist in accurate implant placement during surgery. This personalised approach optimises anatomical fit, potentially improving stability and reducing the risk of complications. 3D printing technology represents a cutting-edge tool that aligns with the principles of precision medicine, offering customised solutions for calcaneal fracture management [50].

New Materials for Fixation

Biodegradable implants: The exploration of biodegradable implants, crafted from materials like polylactic acid, represents a significant advancement in orthopaedic surgery, including calcaneal fracture management. These implants are designed to degrade over time within the body gradually. This innovative approach can eliminate secondary removal surgery, a common requirement with traditional metallic implants. By minimising the risk of long-term complications associated with implant retention, biodegradable implants offer a patient-friendly solution that aligns with sustainability principles and reduces invasiveness [51].

Advanced biocompatible materials: The development of implants using advanced biocompatible materials aims to improve the overall compatibility between the implant and the patient's body. This innovation focuses on reducing the risk of adverse reactions, such as inflammation or rejection, and enhancing the integration of the implant with the surrounding bone tissue. Utilising materials with superior biocompatibility contributes to a more favourable healing environment, potentially lowering the incidence of complications related to immune responses or implant-related inflammation [52].

Nitinol fixation devices: Nitinol, a shape memory alloy composed of nickel and titanium, is gaining attention for its unique mechanical properties. Nitinol fixation devices are being explored for their ability to provide dynamic stabilisation in orthopaedic applications, including calcaneal fractures. Nitinol's distinctive shape memory property allows these devices to adapt to changes in load and temperature, providing a more physiological response to biomechanical stresses. The dynamic nature of nitinol fixation devices may contribute to improved long-term outcomes and enhanced patient comfort by facilitating a more natural response to the forces acting on the calcaneus during weight-bearing activities [53].

Rehabilitation Strategies

Virtual reality (VR) rehabilitation: Integrating VR technology into rehabilitation programs for calcaneal fractures represents a cutting-edge approach to enhance patient engagement and outcomes. VR offers interactive and immersive exercises that simulate real-world scenarios, providing a novel and engaging platform for rehabilitation. By creating a virtual environment tailored to the patient's needs, VR technology encourages active exercise participation, potentially improving compliance and motivation during recovery. This innovative approach not only adds a dynamic element to rehabilitation but also has the potential to accelerate functional recovery and improve overall patient satisfaction [54].

Sensor-based rehabilitation devices: Wearable sensors and smart devices are transforming the landscape of calcaneal fracture rehabilitation by providing real-time monitoring and tracking of patients' movements. These sensors capture data on the range of motion, gait, and other relevant parameters during rehabilitation exercises. The information gathered from these devices can be analysed to customise rehabilitation plans, ensuring that exercises are targeted, effective, and aligned with the individual patient's progress. Integrating sensor-based rehabilitation devices offers a data-driven approach to rehabilitation, optimising outcomes through personalised and adaptive interventions [55].

Tele-rehabilitation services: Integrating Tele-rehabilitation services leverages technology to extend rehabilitation beyond the traditional clinical setting. Patients can access rehabilitation services remotely, enabling ongoing care and monitoring. This approach is precious for individuals in rural or remote locations who may face challenges accessing regular in-person rehabilitation sessions. Tele-rehabilitation facilitates real-time communication between patients and healthcare providers, allowing for remote assessments, exercise guidance, and progress monitoring. This technology-driven approach enhances accessibility and continuity of care, ultimately improving patient outcomes [56].

Biomechanical analysis tools: Advanced biomechanical analysis tools are pivotal in rehabilitating calcaneal fractures by providing detailed insights into gait and movement patterns. These tools enable a thorough biomechanics assessment, allowing healthcare providers to identify specific functional deficits and tailor rehabilitation plans accordingly. By analyzing factors such as joint kinematics, ground reaction forces, and muscle activation patterns, biomechanical analysis tools contribute to a more precise understanding of the patient's movement mechanics. This information informs targeted interventions to address specific challenges, optimising the effectiveness of rehabilitation and promoting a more seamless return to normal activities [57].

Patient education and counselling

Importance of Patient Compliance

Adherence to rehabilitation protocols: Emphasising the importance of strict adherence to prescribed rehabilitation protocols is a cornerstone of successful recovery following calcaneal fractures. Patient compliance with exercises, weight-bearing restrictions, and activity modifications significantly influence the long-term outcomes of fracture management. Reinforcing the rationale behind each aspect of the rehabilitation plan helps patients understand their critical role in their recovery. Clear communication and ongoing support from healthcare providers contribute to improved adherence, facilitating optimal healing and functional restoration [58].

Medication management: Ensuring patients understand the necessity of adhering to medication regimens is vital for adequate pain control and healing. This includes pain management medications and any prescribed anti-inflammatory drugs. Educating patients about the purpose of each medication, potential side effects, and the importance of following the prescribed dosage schedule enhances medication compliance. Addressing patients' concerns or questions about their medications fosters a collaborative approach to pain management, contributing to overall well-being during recovery [59].

Follow-up appointments: Stressing the significance of attending scheduled follow-up appointments with healthcare providers is essential for the comprehensive management of calcaneal fractures. Follow-up appointments allow for ongoing assessment of the healing process, adjustment of treatment plans based on progress, and early identification of potential complications. Regular monitoring enables healthcare providers to address evolving patient needs, ensuring the recovery trajectory remains on course. Clear communication regarding the importance of follow-up appointments encourages patients to participate actively in their ongoing care and promotes optimal long-term outcomes [60].

Weight-bearing restrictions: Communicating the importance of adhering to weight-bearing restrictions, especially during the initial stages of recovery, is critical to prevent strain on the healing calcaneus and to avoid potential setbacks. Patients must understand the rationale behind these restrictions and their impact on the surgical intervention's stability and success. Reinforcing the need for gradual progression from non-weight-bearing to partial and eventually complete weight-bearing aligns with the principles of safe rehabilitation. Clear instructions and ongoing education empower patients to make informed decisions that support the healing process and minimise the risk of complications [61].

Expectations During Recovery

Timeline for healing: Providing patients with a realistic timeline for recovery is essential to managing expectations and fostering informed decision-making. Communicating that the healing process for calcaneal fractures may be gradual and that improvement may continue over an extended period helps patients understand the nature of their recovery. This information empowers patients to navigate rehabilitation with patience and persistence, reducing anxiety and promoting a positive mindset [62].

Functional milestones: Outlining expected functional milestones is crucial for patients to gauge their progress and understand their recovery trajectory. These milestones may include bearing weight, returning to normal activities, and achieving optimal range of motion. Communicating these benchmarks allows patients to track their achievements, motivating adherence to the rehabilitation plan. Clear expectations regarding functional milestones contribute to a sense of accomplishment and provide tangible goals for patients throughout the recovery process [63].

Potential setbacks: Educating patients about potential setbacks is integral to preparing them for the challenges that may arise during recovery. This includes discussing the possibility of temporary increases in pain, challenges during rehabilitation, or variations in progress. Providing this information helps patients mentally and emotionally prepare for the ups and downs of recovery. Additionally, it encourages open communication between patients and healthcare providers, fostering a collaborative approach to overcoming setbacks and optimising outcomes [64].

Return to work and activities: Discussing the anticipated timeline for returning to work and regular activities is crucial for patients to plan their recovery journey effectively. Clear communication about when they can expect to resume daily tasks and work responsibilities allows patients to adjust their lives. This information assists in setting realistic expectations for the pace of recovery, helping patients navigate the transition from rehabilitation to full participation in their usual activities [65].

Lifestyle Modifications

Footwear choices: Advising patients on appropriate footwear is crucial to post-calcaneal fracture care. Recommending shoes that provide adequate support and minimise stress on the foot is essential for optimising the healing process. Encouraging the use of orthopaedic shoes or inserts can provide additional support and comfort during recovery. This guidance helps patients make informed choices that align with their rehabilitation goals and reduce the risk of injury exacerbation through inappropriate footwear [66].

Physical activity guidelines: Discussing modified physical activity guidelines is essential for preventing re-injury and supporting ongoing healing. Patients need clear guidance on avoiding high-impact activities that may strain the healing calcaneus. Gradually reintroducing exercise in a controlled manner helps ensure a progressive return to normal physical activities without compromising the recovery. Personalised recommendations based on the patient's condition contribute to a safe and effective rehabilitation plan [67].

Nutrition and hydration: Emphasising the role of nutrition in bone health and healing is crucial for patients recovering from calcaneal fractures. Encouraging a balanced diet rich in vitamins and minerals essential for bone health, such as calcium and vitamin D, contributes to overall recovery. Adequate hydration is also essential for supporting the body's healing processes. Nutrition education empowers patients to make dietary choices that promote optimal bone healing and overall well-being during recovery [68].

Smoking cessation: For patients who smoke, emphasising the importance of smoking cessation is a vital aspect of comprehensive care. Smoking has been associated with impaired bone healing and an increased risk of complications following fractures. Communicating the potential negative impact of smoking on the healing process motivates patients to make this critical lifestyle modification. Smoking cessation supports calcaneal fracture recovery and improves overall health and well-being [69].

## Conclusions

In conclusion, the comprehensive review of calcaneal fractures underscores the intricacies of their management, encompassing both surgical and non-surgical modalities. Recognising common causes, risk factors, and types of calcaneal fractures provides a foundational understanding essential for accurate diagnosis. Surgical interventions, guided by indications and timing considerations, have evolved with innovations such as minimally invasive techniques, computer-assisted surgery, and novel fixation materials. Concurrently, non-surgical approaches, including rehabilitation strategies and patient education, are pivotal in optimising outcomes. Patient compliance with prescribed protocols emerges as a linchpin for successful recovery, emphasising the importance of diligent postoperative care and long-term follow-up. Recommendations for clinical practice highlight the need for individualised approaches and multidisciplinary collaboration. Future research avenues, spanning biomechanics, regenerative therapies, and telerehabilitation, hold promise in refining current practices and enhancing the overall landscape of calcaneal fracture management. As we reflect on the past and look forward, this comprehensive exploration aims to contribute to the ongoing refinement of clinical strategies, ultimately improving the quality of care and outcomes for individuals grappling with calcaneal fractures.
